# A Novel Workflow for Fast Elucidation of Drug Metabolites for Screening—Combining In Silico Metabolite Prediction With Trapped Ion Mobility QTOF‐MS

**DOI:** 10.1002/dta.70062

**Published:** 2026-04-22

**Authors:** Annette Zschiesche, Birgit Schneider, Ilona Nordhorn, Carsten Baessmann, Laura M. Huppertz, Jürgen Kempf

**Affiliations:** ^1^ Institute of Forensic Medicine, Forensic Toxicology, Medical Center—University of Freiburg Faculty of Medicine, University of Freiburg Freiburg Germany; ^2^ Hermann Staudinger Graduate School University of Freiburg Freiburg Germany; ^3^ Bruker Daltonics GmbH & Co. KG Bremen Germany

**Keywords:** biomarkers, CCS, in silico prediction, PASEF, trapped ion mobility spectrometry

## Abstract

Urine is one of the preferred matrices for standard toxicological analysis, which makes the inclusion of drug metabolites in targeted and untargeted screening mandatory. Mass spectrometry is key for substance identification, but updating methods for emerging substances like new psychoactive substances (NPS) is challenging due to the limited availability of reference standards for metabolites. This is particularly problematic for drugs that are barely or not detectable at all in urine. Insufficient metabolic knowledge and lack of spectral data carry the risk of false negatives.

This study evaluates a non‐targeted workflow using ultrahigh‐performance liquid chromatography‐trapped ion mobility spectrometry time‐of‐flight mass spectrometry (UHPLC‐timsTOF‐MS) and dedicated processing software (MetaboScape), integrating in silico metabolite prediction (BioTransformer), fragmentation (MetFrag), collision cross‐section (CCS) prediction, and library searching. Quetiapine was selected as a model compound. Phase I metabolites were generated via pooled human liver microsomes (pHLMs) and analyzed by UHPLC‐timsTOF‐MS. Features were extracted and annotated with MetaboScape. The workflow successfully annotated 20 phase I metabolites in the pHLM assay, with 13 confirmed by library matching and 18 by BioTransformer. These metabolites were added to a targeted UHPLC‐QTOF‐MS method for analysis of 30 quetiapine‐positive ante‐ and post‐mortem urine samples from forensic casework. This revealed *N*‐, *O*‐dealkyl and carboxylated metabolites as the most abundant biomarkers in human urine.

This integrated approach enables rapid and reliable metabolite detection, supports biomarker discovery, and facilitates routine screening updates, especially for substances without reference standards. Although not intended for exhaustive metabolic characterization, it offers practical applicability in evolving drug landscapes.

## Graphical abstract

UHPLC‐timsTOF‐MS with in silico prediction and automated annotation enabled rapid identification of quetiapine metabolites from pHLMs, facilitating their inclusion in targeted LC‐QTOF‐MS urine screening and improving biomarker detection for toxicological applications.

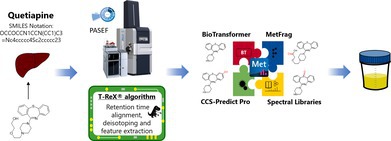

## 1 Introduction

According to data from the EU Early Warning System, approximately 50 new psychoactive substances (NPS) continue to emerge in the European Union each year [1]. Screening for these compounds in urine can be quite challenging. Cross‐reactivities of immunochemical methods can vary from good (e.g., benzodiazepines) [2] to insufficient (e.g., synthetic cannabinoids) [3]. Mass spectrometry (MS) is the gold standard for identification of compounds in a standard toxicological analysis [4]. But whether used for initial screening or as a confirmatory analysis alongside other methods, MS approaches require regular updates. However, reference substances for drug metabolites are usually not commercially available, or only to a limited extent. This is particularly a problem with substances whose parent compound is hardly or not at all detectable in urine. Missing knowledge about metabolism and spectral information of metabolites may lead to false negative findings. To cope with this multitude of new substances and their detection, including metabolites, the use of in silico tools to predict data is increasing [5, 6, 7, 8, 9].

If the parent compound is available, pooled human liver microsome (pHLM) assays are an easy‐to‐use, animal‐free methodology to generate phase I metabolites in vitro, which can show good agreement within the metabolic profile found in human urine samples [6, 9, 10, 11, 12].

A non‐targeted workflow using UHPLC‐timsTOF‐MS combined with sophisticated software tool can be used to identify features of in silico generated metabolites. MetaboScape [13] combines the in silico metabolite prediction tool BioTransformer [14, 15], MetFrag [16, 17] for in silico fragmentation, collision cross section (CCS) prediction using CCS‐Predict Pro 2024 [18], an algorithm that predicts CCS values from 2D compound structures, and the use of commercial (e.g., Maurer, Meyer, Helfer, and Weber (MMHW) [19]) or in‐house generated spectral libraries. Retention time alignment, deisotoping and feature extraction was performed by the implemented T‐ReX 4D algorithm (Time aligned Region complete eXtraction). Ion mobility mass spectrometry (IMS‐MS) is widely used in metabolomics, lipidomics and proteomics [20, 21]. With IMS‐MS, alongside mass‐to‐charge ratio, fragment ions, and retention time, a “fourth dimension” is created that enhances confidence in compound identification [22, 23]. Sensitive and rapid MS/MS spectra were obtained by using Parallel Accumulation Serial Fragmentation (PASEF), a fast acquisition mass spectrometry technique combining trapped ion mobility and time‐of‐flight separation. In parallel accumulated ions within a trapped ion mobility device are sequentially released to the mass analyzer for fragmentation. This process multiplies the number of MS/MS spectra acquired per second, significantly increasing sequencing speed without loss of sensitivity and provides CCS values. It is especially efficient for complex samples and detection of low‐abundance analytes [24]. Retention time, principle ion and fragment ions of detected metabolites can be transferred to existing routine screening methods, e.g., the compound database of the TargetScreener HR (Bruker Daltonics software for high‐resolution mass spectrometry (HRMS) screening, see Section 2). While HRMS instruments are technically mature, their untargeted (metabolite) identification potential remains underutilized in routine forensics [25, 26, 27, 28, 29].

Quetiapine, a well‐studied and widely used atypical antipsychotic medication approved in 1997 by the Food and Drug Administration (FDA) to treat various mental health conditions, was used as a model substance to demonstrate the suitability of the workflow described above for the subsequent analysis of real positive urine samples. The neuroleptic quetiapine, with a dibenzothiazepine core moiety, is extensively metabolized primarily in the liver, yielding multiple well‐known metabolites. Norquetiapine (*N*‐desalkylquetiapine), the most active metabolite of quetiapine, is primarily formed by CYP3A4, whether CYP2D6 plays a role, particularly in the formation of the active metabolite 7‐hydroxy‐*N*‐desalkylquetiapine [30, 31, 32, 33]. Intoxications, as well as death cases linked to quetiapine overdoses, were reported [34, 35, 36, 37].

For this purpose of establishing a proof of concept for the integrated in silico‐assisted workflow, quetiapine metabolites were generated in a pHLM assay and analyzed using a non‐targeted approach with the timsTOF Pro2 in PASEF mode. Authentic urine samples that tested positive for quetiapine intake with an Impact II QTOF were reprocessed including the previously identified quetiapine metabolites to find the most abundant in vivo biomarkers for quetiapine uptake.

## 2 Materials and Methods

Quetiapine was purchased by TRC (Toronto, Canada). Morphine‐*D3*, haloperidol‐*D4*, MDMA‐*D5*, and diazepam‐*D5* were obtained by LGC (Teddington, UK), whereas risperidone‐*D4* was obtained by Cerilliant (Round Rock, TX, USA).

Acetonitrile (ACN, LC‐MS grade), ammonium formate 10 M (99.995%), were bought from Sigma‐Aldrich (Steinheim, Germany). Formic acid (Rotipuran ≥ 98%, p.a.), sodium hydroxide (≥ 99%, p.a.), and methanol (MeOH) were obtained from Carl Roth (Karlsruhe, Germany). Isopropanol (Prepsolv) was obtained from Merck (Darmstadt, Germany). Deionized water was prepared using a Medica Pro deionizer from ELGA (Celle, Germany).

Pooled human liver microsomes (pHLMs, 150 donors, 20 mg/mL protein in 250 mM sucrose) and NADPH‐regenerating solutions A (26 mM NADP^+^, 66 mM glucose‐6‐phosphate, and 66 mM MgCl_2_ in H_2_O) and B (40 U/mL glucose‐6‐phosphate dehydrogenase in 5 mM sodium citrate) with a reductase activity of 0.43 μmol/min × mL were obtained from Corning (Amsterdam, the Netherlands). Potassium phosphate buffer (0.5 M, pH 7.4) was also sourced from the same supplier.

Blank urine samples were donated by two volunteers and were tested prior analysis for absence of quetiapine and its metabolites.

Eluent C consists of H_2_O/MeOH (99:1) with 5 mM ammonium formate and 0.01% formic acid, eluent D of MeOH with 5 mM ammonium formate and 0.01% formic acid. Eluents were freshly prepared prior analysis.

A sodium formate clusters solution was prepared for both external instrument calibrations. The mixture combined 500 mL deionized water, 500 mL isopropanol, 2 mL formic acid, and 10 mL of sodium hydroxide (1 M).

### 2.1 Pooled Human Liver Microsomes Incubations (pHLMs)

For phase I metabolite identification and plausibility control, pHLM assays were performed according to an already published procedure [38]. Briefly, 1 μL of a quetiapine stock solution (1 mg/mL in MeOH) was added to a reaction mixture consisting of 5 μL of pHLM, NADPH‐regenerating solution A (5 μL) and B (1 μL), 20 μL of phosphate buffer (0.5 M, pH 7.4) and 68 μL of deionized water. The incubation was conducted at 37°C for 30 min. The mixture was quenched afterwards by adding 100 μL of ice‐cold acetonitrile and 50 μL of an ammonium formate solution (10 M). After centrifugation (10 min, 16,110 × g), 30 μL of the supernatant was evaporated to dryness under a stream of nitrogen and reconstituted in 30 μL of C/D (*v/v* = 50/50). A negative control (no quetiapine) and a substance control (no pHLM) were performed additionally. The samples of the pHLM assays were analyzed using the Bruker timsTOF Pro 2 (TargetScreener 4D) and the QTOF Impact II (TargetScreener HR). No internal standards were used because the experiments were designed to measure relative metabolite formation. In this design, the peak areas of the metabolites were normalized to the peak area of the main metabolite.

### 2.2 Human Urine Samples

In total, data of *n* = 30 human urine samples (ante‐mortem: *n* = 20, post‐mortem: *n* = 10) was reprocessed retrospectively with the updated TASQ method. Samples used in this study were found quetiapine‐positive in routine LC‐QTOF‐MS screening (Impact II QTOF, Bruker Daltonics, Bremen, Germany) at the Institute of Forensic Medicine in Freiburg, Germany before. Samples were anonymized and derived from forensic toxicology casework (e.g., abstinence control, criminal investigations, clinical intoxication cases, and medicolegal death investigations) and were stored at −20°C prior to analysis. An aliquot of 0.1 mL of each urine was used. The samples were fortified with 5 μL of internal standard mix consisting of morphine‐*D3*, haloperidol‐*D4*, risperidone‐*D4*, MDMA‐*D5*, and diazepam‐*D5*. The used forensic accredited LC‐QTOF‐MS routine screening method uses a mix of four deuterated internal standards to cover a wide retention time and polarity range.

The liquid–liquid extraction was performed by adding 0.5 mL of ice‐cold acetonitrile, shaking for 5 min, followed by centrifugation (10 min, 16,110 × g). The organic phase was evaporated to dryness under a stream of nitrogen. The residue was resolved in 25 μL of the eluent consisting of C/D (*v/v* = 50/50).

The TargetScreener HR [39] TASQ processing method was expanded to include quetiapine and its metabolites based on the results of pHLM incubations in combination with IMS‐MS. For data analysis, the peak areas of the metabolites in each urine sample were normalized to that of the most abundant metabolite.

### 2.3 Ultrahigh‐Performance Liquid Chromatography

Both methods (TargetScreener 4D and TargetScreener HR) used the same LC system with the same parameters. An Elute UHPLC system was operated by HyStar ver. 6.0 (both: Bruker Daltonics, Bremen, Germany). Injection volume was 2 μL and a Bruker Intensity Solo C18–2 (1.8 μm, 2.1 × 100 mm) column with an appropriate column guard at 40°C was used. The gradient and flow rate were composed as follows: Starting at 4% D for 0.1 min (0.2 mL/min), increasing to 18.3% D at 1 min (0.2 mL/min), to 50.0% D at 2.5 min (0.223 mL/min), and to 99.9% D at 14 min (0.4 mL/min). The gradient was held for 2 min at 99.9% D (0.48 mL/min), decreasing to 4% D at 16.1 min, keeping the flow rate at 0.48 mL/min. The gradient and flow rate were held for 2.9 min, and after 0.1 min, the flow rate was decreased to 0.2 mL/min, keeping 4% D constantly for a further 0.9 min. The total run time was 20 min.

### 2.4 Trapped Ion Mobility Quadrupole Time‐of‐Flight Mass Spectrometry—TargetScreener 4D

The timsTOF Pro 2 equipped with a VIP‐HESI source (Vacuum Insulated Probe Heated Electrospray Ionization), both from Bruker Daltonics, Bremen, Germany, was used. The resolution was 40,000 for the measured mass range. The mass spectrometer was operated in positive mode, within a mass range *m/z* 20–1300 with the following TIMS settings: 1/K_0_: 0.1–1.5 V·s/cm^2^, ramp time: 150 ms. Nebulizer: 3.0 bar, dry gas with a flow of 10.0 L/min and a temperature of 220°C. The sheath gas temperature was set to 470°C and the flow to 4.0 L/min, respectively. High‐purity nitrogen was used for both the dry gas and the sheath gas. The PASEF (ver. 5.0.4.0, Bruker Daltonics, Bremen, Germany) settings can be found in Table S1.

Data processing was carried out using MetaboScape ver. 2024b and the implemented CCS‐Predict Pro ver. 2024 (both: Bruker Daltonics, Bremen, Germany). The settings of MetaboScape and the implemented BioTransformer application can be found in Tables S2 and S3, respectively.

The results were incorporated into the TASQ method (ver. 2024b, Bruker Daltonics, Bremen, Germany) for the TargetScreener HR (Bruker Daltonics, Bremen, Germany) when the feature was annotated and predicted and was not present in the control samples. This included the sum formula of the molecular ion (∆*m/z* ≤ 5 ppm), the CCS value (≤ 5%), the matching MS/MS spectra with MetFrag prediction (MS/MS score: > 900) and the signal‐to‐noise ratio: > 3. The sum formula of the molecular ion, the *m/z* of the qualifier ions, and the retention time were implemented. The TASQ method can be found in Table S4.

### 2.5 Heated Electrospray Ionization Quadrupole Time‐of‐Flight Mass Spectrometry (HESI‐QTOF‐MS)—TargetScreener HR

The Impact II QTOF mass spectrometer (resolution: 40,000 for *m/z* 30–1000) was equipped with a VIP‐HESI source (Bruker Daltonics, Bremen, Germany) and was operated in positive mode using Bruker's TargetScreener HR‐method. The instrument was operated in broadband collision‐induced dissociation (bbCID) mode (collision energy: 30 eV, collision energy spread: 6 eV, scanning range: *m/z* 30–1000) within an acquisition rate of 2 Hz. The end plate offset was set to 500 V, capillary voltage: 2500 V, nebulizer gas: 2.5 bar; dry gas and temperature 8.0 L/min and 230°C, respectively, probe gas 5.0 L/min, and probe gas temperature 400°C. Data Analysis (ver. 5.6) and TASQ ver. 2024b (both: Bruker Daltonics, Bremen, Germany) were used for data processing. Subsequent data handling was performed using Microsoft Excel 2016 (Microsoft Corporation, Redmond, WA, USA).

## 3 Results and Discussion

### 3.1 Metabolites of Quetiapine In Vitro (pHLM)—TargetScreener 4D

As reported by several studies, the tetracyclic compound quetiapine (Q0) undergoes extensive metabolism [8, 31, 40]. The metabolites found in the pHLM assay annotated by BioTransformer and the spectral library MMHW (ver. 2017) with the experimentally determined and predicted CCS values can be found in Table 1.

**TABLE 1:** Quetiapine and annotated phase I metabolites using UHPLC‐timsTOF and MetaboScape.

	Feature									Spectral library (SL)				BioTransformer (BT)			
MetID	Molecular formula	Annotation		theor. *m/z*	t_R_/min	CCS	∆*m/z*/ppm	mSigma	Rel. Int./ %	Name	MS/MS score	CCS predicted	∆CCS/ %	Name (Nr. of proposed structures)	MS/MS score	CCS predicted	∆CCS/ %
**Q0** (Quetiapine)	**C**_**21**_ **H**_**25**_ **N**_**3**_ **O**_**2**_ **S**	**SL**	**BT**	384.1707	7.48	193.5	−0.33	16	33.65	Quetiapine	958	189.9	1.90	Quetiapine	959	189.9	1.90
**Q1** (Norquetiapine)	**C**_**17**_ **H**_**17**_ **N**_**3**_ **S**	**SL**	**BT**	296.1216	7.19	168.8	−1.34	26	16.10	Quetiapine‐M (*N*‐dealkyl‐)	978	167.9	0.53	Quetiapine – C_4_H_8_O_2_ (1)	966	167.9	0.53
**Q2**	**C**_**17**_ **H**_**17**_ **N**_**3**_ **OS**	—	**BT**	312.1165	9.73	172.5	−1.88	37	1.41	N/A	N/A	N/A	N/A	Quetiapine – C_4_H_8_O (9)	981	171.2	0.74
**Q3** (7‐Hydroxynorquetiapine)		**SL**	**BT**		4.87	174.2	−0.69	6.0	0.23	Quetiapine‐M (*N*‐dealkyl‐OH)	892	175.8	*−0.91*	Quetiapine – C_4_H_8_O (9)	972	171.1	1.80
**Q4** (Norquetiapine Sulfoxide)		**SL**	**BT**		5.24	172.3	−0.06	26	0.99	Quetiapine‐M (*N*‐dealkyl‐sulfoxide)	932	171.0	0.75	Quetiapine – C_4_H_8_O (9)	942	170.4	1.10
**Q5** (*O*‐Desalkylquetiapine)	**C**_**19**_ **H**_**19**_ **N**_**3**_ **OS**	—	**BT**	338.1322	9.96	181.4	−1.00	2.3	0.02	N/A	N/A	N/A	N/A	Quetiapine – C_2_H_6_O (1)	867	178.7	1.50
**Q6**	**C**_**19**_ **H**_**21**_ **N**_**3**_ **OS**	—	**BT**	340.1478	7.35	183.2	0.21	3.5	13.76	N/A	N/A	N/A	N/A	Quetiapine – C_2_H_4_O (1)	954	179.6	2.00
**Q7**	**C**_**19**_ **H**_**19**_ **N**_**3**_ **O**_**2**_ **S**	**SL**	**BT**	354.1271	7.94	184.8	−0.43	6.1	1.83	Quetiapine‐M (N‐CH2‐COOH)	921	181.9	1.62	Quetiapine – C_2_H_6_ (12)	982	181.4	1.90
**Q8**	**C**_**19**_ **H**_**21**_ **N**_**3**_ **O**_**2**_ **S**	—	**BT**	356.1427	4.92	188.3	−0.56	9.0	0.20	N/A	N/A	N/A	N/A	Quetiapine – C_2_H_4_ (11)	1000	182.5	3.20
**Q9**		**SL**	**BT**		8.07	187.5	−0.46	6.6	1.00	Quetiapine‐M (*O*‐dealkyl‐sulfoxide)	552	182.3	2.86	Quetiapine – C_2_H_4_ (11)	961	182.0	3.00
**Q10**		**SL**	**BT**		5.37	179.9	−0.16	13	0.30	Quetiapine‐M (*O*‐dealkyl‐sulfoxide)	933	182.3	−1.30	Quetiapine – C_2_H_4_ (11)	965	182.5	−1.40
**Q11**		**SL**	**BT**			186.8				Quetiapine‐M (*O*‐dealkyl‐sulfoxide)	957	182.2	2.50	Quetiapine – C_2_H_4_ (11)	976	182.1	2.60
**Q12**	**C**_**19**_ **H**_**19**_ **N**_**3**_ **O**_**3**_ **S**	**SL**	**—**	370.122	8.61	188.6	−0.82	18	2.86	Quetiapine‐M (N‐CH2‐COOH‐piperazine)	842	—	—	N/A	N/A	N/A	N/A
**Q13**		**SL**	**—**		5.7	188.2	−0.44	18	0.05	Quetiapine‐M (N‐CH2‐COOH‐sulfoxide)	894	—	—	N/A	N/A	N/A	N/A
**Q14** (Quetiapine carboxylic acid)	**C**_**21**_ **H**_**23**_ **N**_**3**_ **O**_**3**_ **S**	**—**	**BT**	398.1533	7.82	195.5	−0.07	9.9	0.01	N/A	N/A	N/A	N/A	Quetiapine + O – H_2_ (12)	983	191.9	1.90
**Q15** (7‐Hydroxyquetiapine)	**C**_**21**_ **H**_**25**_ **N**_**3**_ **O**_**3**_ **S**	**SL**	**BT**	400.1689	5.56	198.5	−0.31	5.3	2.22	Quetiapine‐M (HO‐)	918	197.7	*0.40*	Quetiapine + O (11)	948	192.0	3.40
**Q16** (Quetiapine sulfoxide)		**SL**	**BT**		5.06	197.4	−0.02	15	18.65	Quetiapine‐M (sulfoxide)	935	192.4	2.60	Quetiapine + O (11)	975	191.8	2.90
**Q17**	**C**_**21**_ **H**_**25**_ **N**_**3**_ **O**_**4**_ **S**	**SL**	**BT**	416.1639	6.13	196.4	−0.41	15	6.30	Quetiapine‐M (di‐HO‐)	664	205.4	*−4.40*	Quetiapine + O_2_ (58)	908	193.9	1.30
**Q18**		**—**	**BT**		4.49	202.1	−0.13	13	0.10	N/A	N/A	N/A	N/A	Quetiapine + O_2_ (57)	978	194.5	3.90
**Q19**		**—**	**BT**		5.39	202.9	−0.12	16	0.21	N/A	N/A	N/A	N/A	Quetiapine + O_2_ (58)	960	195.1	4.00
**Q20**		**SL**	**BT**		5.96	202.1	0.96	15	0.09	Quetiapine‐M (di‐HO‐)	694	205.4	*−1.60*	Quetiapine + O_2_ (58)	864	194.7	3.80

*Note:* ∆CCS [%] in *italics*: The MMHW Library does not specify the structure clearly. The functional group is shown near the molecule but not assigned to a defined position, making reliable CCS value prediction infeasible.

Abbreviations: ∆CCS, difference between predicted and measured CCS values in %; ∆*m/z*, difference between theoretical and measured *m/z* in ppm; BT, BioTransformer; CCS, collusion cross section; MS/MS Score, match factor between predicted (BT) or the spectral library MMHW (SL) and measured MS/MS spectra. 100% match is a score of 1000; mSigma, value for the quality of the isotope pattern; N/A, not available; Rel. Int., relative intensity of a metabolite to the sum of all metabolite intensities in %; SL, spectral library; t_R_, Retention time.

The TargetScreener 4D approach was able to annotate 20 phase I metabolites in the pHLM assay, with 13 confirmed by library matching (MMHW) and 18 phase I biomarkers with BioTransformer. MMHW annotated two metabolites exclusively, whereas BioTransformer annotated seven metabolites exclusively. In all cases, the parent compound quetiapine (Q0) was annotated in the pHLM assay as well (overall relative intensity: 33.65%). Gunter et al. experimentally determined a CCS value of 191.23 Å^2^ for quetiapine [M + H]^+^ [41]. In this study, the experimentally determined CCS value for the same adduct was 193.5 Å^2^. The deviation between the experimentally determined CCS values is 1.19% and can be considered low. Hines et al. experimentally determined a value of 191.5 Å^2^ for the hydrogen adduct [42]. Mollerup et al. predicted a CCS value [M + H]^+^ for quetiapine of 192.9 Å^2^ using artificial neural networks [43]. CCS‐Predict Pro revealed a CCS value of 189.9 Å^2^ for the same species. The DrugBank gave a predicted CCS value for the [M + H]^+^ of quetiapine of 191.6 Å^2^ [44, 45]. Differences in prediction of CCS values lie in several factors. Different prediction models differ in size and diversity of the training set [46], some tools use 2D‐based structures, e.g., SMILES (Simplified Molecular Input Line Entry System), and others 3D models, gaining more accurate results [47]. But also, prediction models based on experimental CCS data are influenced by the instrument types used [48].

Norquetiapine (Q1, Quetiapine ‐C_4_H_8_O_2_), the major active metabolite of quetiapine and also known as *N*‐desalkylquetiapine, was detectable in all urine samples analyzed by the TargetScreener HR as the most abundant metabolite as well as in vitro in the pHLM assay [31]. Q1 showed with an experimental CCS value of 168.8 Å^2^ a lower value than quetiapine (Q0) with 193.5 Å^2^ due to the smaller molecule by the loss of the *N*‐alkyl group and the associated smaller effective cross section. CCS values are generally increasing with increasing molecular mass as can be seen in Table 1.

Most isomeric quetiapine metabolites (e.g., Q3, Q4, Q8, and Q9) were readily distinguished by their retention times alone (Table 1). Notably, Q10 and Q11 coeluted at identical retention times but could be unambiguously differentiated by their distinct CCS values (179.9 vs. 186.8 Å^2^). This example demonstrates the value of incorporating IMS into the 4D workflow, as conventional LC‐QTOF‐MS would have detected these as a single peak, potentially leading to their assignment as one metabolite rather than two distinct structural isoforms. This example illustrates how the 4D approach (*m/z*, t_R_, CCS, and MS/MS) enhances metabolite annotation confidence, particularly for complex phase I transformation mixtures where chromatographic baselines may limit resolution. In general unknown screening, CCS prediction also provides higher accuracy in the annotation of metabolites. Ion mobility also makes it possible to obtain cleaner MS/MS spectra. Thus, integrating orthogonal IMS data strengthens structural assignments across the dataset and provides a scalable framework for broader applications in forensic toxicology.

In general, the metabolites detected in vitro were consistent with previously published results of metabolites formed in pHLMs [49].

### 3.2 Metabolites of Quetiapine In Vivo (Urines) and In Vitro (pHLM)—TargetScreener HR

With the help of the results of the pHLM assay, a TASQ‐method was developed for detecting quetiapine uptake in urine samples without using ion mobility. The metabolites found in ante‐mortem (am) and post‐mortem (pm) urine are shown as a heat map in Table 2. The peak areas of each biomarker were normalized to the most intense metabolite of each sample.

**TABLE 2:**
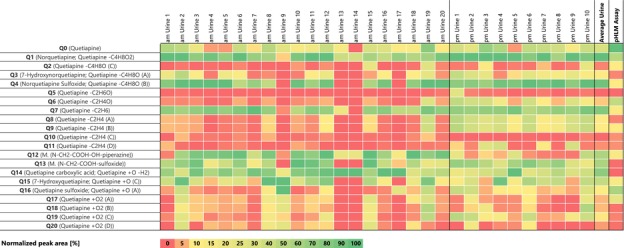
LC‐QTOF‐MS findings in ante‐mortem (am) and post‐mortem (pm) human urine samples using the adopted TargetScreener HR TASQ method.

**FIGURE 1:**
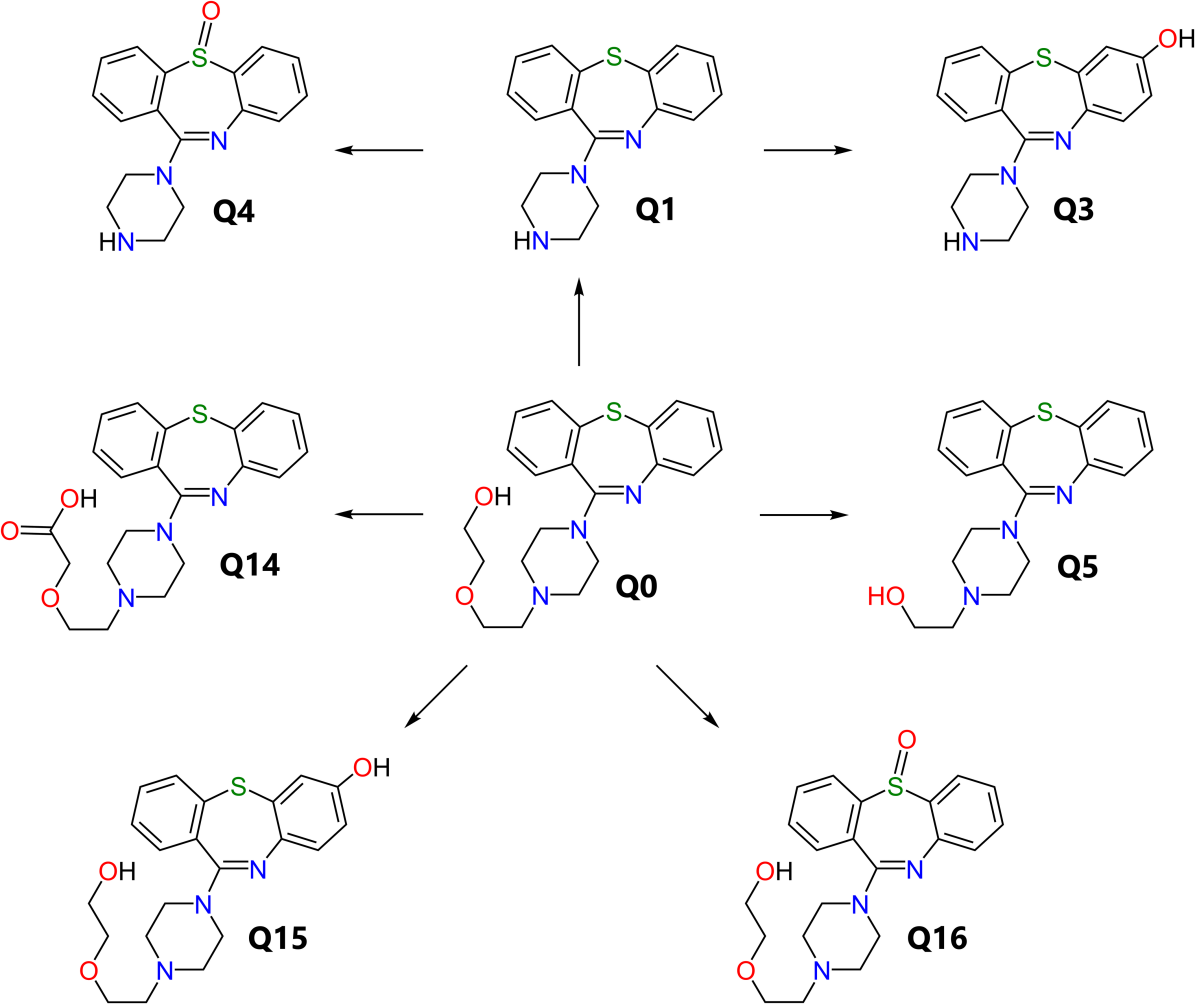
The main metabolites of quetiapine (Q0) found in vivo.

Figure 1 presents the main metabolites of quetiapine in human urine samples detected.

The parent substance quetiapine (Q0) could be detected in nearly all urine samples as it was seen in the pHLM assay. Quetiapine has an elimination half‐life of approximately 6–7 h and is mainly excreted by the kidneys (approx. 73%) [40]. The larger the peak area of quetiapine, the more metabolites tended to be detected in urine. Reanalyzed urine samples from forensic cases with known quetiapine uptake showed mostly congruent results in the metabolite spectrum. Norquetiapine (Q1, Quetiapine ‐C_4_H_8_O_2_) and in contrast to the findings in the pHLM assay, the carboxylic acid metabolite (Q14, Quetiapine +O ‐H_2_) showed high signal intensities in post‐ and ante‐mortem urine samples. According to the literature, approximately 30% of quetiapine is converted to the carboxylic acid metabolite during biotransformation processes in the human organism [50]. The heat map (Table 2) partially reflects this statement based on normalized peak areas. This biomarker is particularly recommended for screening for low doses of quetiapine, as traditional drug tests only detect quetiapine itself and 7‐hydroxyquetiapine, which can lead to false negative results in adherence tests in patients [51]. Q14 is built by oxidation of the ethoxyethanol sidechain. Other high abundant metabolites derive from sulfoxidation resulting in quetiapine sulfoxide (Q16, Quetiapine +O), hydroxylation (Q15, Quetiapine +O), or combinations of the latter (Q3; Quetiapine ‐C_4_H_8_O (A), Q4: Quetiapine ‐C_4_H_8_O (B)).

Q8 (Quetiapine ‐C_2_H_4_ (A)) and Q9 (Quetiapine ‐C_2_H_4_ (B)) appear to be elevated in post‐mortem samples. The reason for this is unclear. However, the data suggest that the same biomarkers can also be used for post‐mortem urine samples.

Though some quantitative differences were observed, particularly for Q12, Q13, and Q14, the comparison of average normalized peak areas between routine samples (average urine) and the pHLM assay was mostly consistent. The analysis of a certain number of authentic samples is therefore essential for the proper selection of suitable biomarkers.

Based on the peak areas, quetiapine (Q0) itself and the main metabolites norquetiapine (Q1), norquetiapine sulfoxide (Q4), and the carboxylic acid metabolite (Q14) are recommended for routine LC‐QTOF‐MS screening and were added to the TargetScreener TASQ database for this purpose.

### 3.3 Limitations

In general, in vitro assays, e.g., pHLMs, mostly cannot reproduce the full metabolism of the complex human organism, e.g., by lacking many cytosolic enzymes. Therefore, in addition to quantitative differences, qualitative differences between pHLM findings and data from human samples are to be expected [6, 52, 53, 54]. Although human specimens show the complete metabolic pattern with all its variants, there is also a major drawback to using samples from case work. There is usually no information about important details such as the dosage, time of the last intake or, in the case of post‐mortem samples, the chronological context between intake, time of death, and sampling [6, 9]. Also the consumption of antagonists could lead to changed metabolic spectra. Variations in metabolite peak areas can result from individual differences in enzyme expression, particularly with regard to quetiapine and CYP2D6 activity [55]. Additionally, ranking by peak areas does not necessarily reflect actual metabolite concentrations possibly due to different ionization efficiencies and matrix effects.

Furthermore, the urine samples were not hydrolyzed but only phase I metabolites were predicted within this workflow. In this setting, pHLM can only generate phase I metabolites. To produce the respective glucuronides, uridine diphosphate‐glucuronic acid, and alamethicin must be added [6, 56]. Biomarkers of consumption could conjugate with glucuronic acid as shown for quetiapine and its metabolite norquetiapine in vitro in HepaRG cells by Le Daré et al. [31]. Nevertheless, Strickland et al. stated that phase I metabolites were the main urinary markers [51].

Some metabolites, such as Q13 (Quetiapine‐M (N‐CH_2_‐COOH‐sulfoxide)), could only be annotated because the corresponding reference spectra were available in the MMHW library (2017). However, Q13 is not among the main or diagnostically relevant metabolites of quetiapine. This reflects a general limitation of the workflow. The accuracy of metabolite annotation depends on the coverage and quality of both spectral libraries and biotransformation prediction tools. Only a few metabolites – none of which represent primary consumption markers – could not be predicted by BioTransformer or located in the spectral library. In these cases, the lack of a structural proposal and corresponding SMILES representation prevented CCS determination. Nevertheless, documenting these gaps is an essential part of the study, as it underscores current limitations in automated biotransformation prediction and spectral reference coverage for emerging drugs. By identifying where existing tools are inadequate, this study establishes a basis for improving future metabolite annotation and identification workflows in forensic and toxicological research. If other in silico tools, such as GLORYx [57], SyGMa [58], or MetaPredictor [59], would be used additionally, the spectrum of annotated metabolites could probably be extended [6].

## 4 Conclusions

The use of pHLMs, UHPLC‐timsTOF‐MS, and a combination of sophisticated software tools in MetaboScape to detect metabolites of the model compound quetiapine after biotransformation prediction and in silico fragmentation of potential metabolites was demonstrated successfully. It allowed fast annotation and review of drug metabolites in a single, multifaceted analysis, with good agreement with known data from the literature and spectral libraries. Obtained metabolic information was used to adapt our LC‐QTOF‐MS screening approach and retrospectively screen data from quetiapine‐positive urine samples from routine case work.

This user‐friendly, untargeted approach enables the identification of biomarkers and provides essential feature data for establishing a mass spectrometry‐based screening protocol for these metabolites in human urine samples. Although this workflow offers significant assistance for, e.g., forensic toxicologists, it is not designed for comprehensive metabolic characterization of compounds.

Spectral data from pHLM assays can be used for future routine screening with traditional targeted methods. Analysis of real urine samples can help to identify the most abundant metabolites in vivo. This workflow also allows retrospective reprocessing of existing bbCID data using the updated TASQ database.

Following this successful proof of concept, this workflow will be used for timely identification of suitable biomarkers for uptake of emerging NPS, especially new synthetic cannabinoids.

## Funding

The authors have nothing to report.

## Conflicts of Interest

I.N., B.S., and C.B. are employed by Bruker Daltonics, Bremen, Germany, which is a supplier of commercial MS instruments. The authors do not report any conflicts of interest.

## Supporting information


**Table S1:** PASEF settings.
**Table S2:** Settings of spectral library (MMHW, ver. 2017) in MetaboScape.
**Table S3:** Settings of BioTransformer.
**Table S4:** The adopted TASQ‐Method.

## Data Availability

The data that support the findings of this study are available in (Table S1: PASEF settings; Table S2: Settings of Spectral Library (MMHW) in MetaboScape; Table S3: Settings of BioTransformer; Table S4: Adopted TASQ Method) of this article.
