# Supplementary material for: A Novel Workflow for Fast Elucidation of Drug Metabolites for Screening—Combining In Silico Metabolite Prediction With Trapped Ion Mobility QTOF‐MS
Source: Drug Test Anal. 2026 Apr 22;18(7):841–51. doi: 10.1002/dta.70062 (PMC13327197; doi:10.1002/dta.70062)
Supplement: Supplementary file 1 — Table S1: PASEF settings. Table S2: Settings of spectral library (MMHW, ver. 2017) in MetaboScape. Table S3: Settings of BioTransformer. Table S4: The adopted TASQ‐Method. [file DTA-18-841-s001.docx]

**A Novel Workflow for Fast Elucidation of Drug Metabolites for Screening – Combining *In Silico* Metabolite Prediction with Trapped Ion Mobility QTOF-MS**

Annette Zschiesche^1,2*^, Birgit Schneider^3^, Ilona Nordhorn^3^, Carsten Baessmann^3^, Laura M. Huppertz^1^, Jürgen Kempf^1^

^1^Institute of Forensic Medicine, Forensic Toxicology, Medical Center – University of Freiburg, Faculty of Medicine, University of Freiburg, Albertstr. 9, 79104 Freiburg, Germany

^2^Hermann Staudinger Graduate School, University of Freiburg, Hebelstr. 27, 79104 Freiburg, Germany

^3^Bruker Daltonics GmbH & Co. KG, Fahrenheitstraße 4, 28359 Bremen, Germany

**Table S1: PASEF settings**

| Precursor Ions | Number of PASEF ramps: 2  Total cycle time: 0.97 s  Charge Minimum: 0  Charge Maximum: 1 |
| --- | --- |
| Scheduling | Precursor Repetitions: Simple  Target Intensity: 10000  Intensity Threshold: 100 |
| Active Exclusion | Yes  Release after 0.1 min |
| Collision Energy Settings | Collision Energy 1: 24.0 eV  Collision Energy 2: 36.0 eV  Collision Energy 3: 50.0 eV |
| Isolation Width Settings | Mass [*m/z*] 50, Width [*m/z*] 2.0  Mass [*m/z*] 1000, Width [*m/z*] 6.0 |
| Stepping | TIMS Stepping, 1/K_0_ Range: 0.10 – 1.50 Vs/cm² |

**Table S2: Settings of Spectral Library (MMHW, ver. 2017) in MetaboScape**

| Tolerances and Scoring: | *m/z*: 2.0 – 5.0 ppm  mSigma: 20 – 250  MS/MS score: 400 – 800  CCS: 3.0 – 10.0% |
| --- | --- |

**Table S3: Settings of BioTransformer**

| SMILES quetiapine: | OCCOCCN4CCN(C2=NC1=CC=CC=C1SC3=CC=CC=C23)CC4 |
| --- | --- |
| Metabolic transformation: | Phase I (CYP450) Transformation, Number of reaction steps to calculate: 2 steps |
| Tolerances and Scoring: | *m/z*: 3.0 – 5.0 ppm  mSigma: 15 – 200  MS/MS score: 300 – 900  CCS: 2.0 – 10.0% |

**Table S4: The adopted TASQ-Method**

| **M meas.** | ***m/z*** | **t_R_ min** | **Ions** | **1/K_0_** | **Formula** | **Name** | **Qual1** | **Qual2** | **Qual3** | **Qual4** | **Qual5** |
| --- | --- | --- | --- | --- | --- | --- | --- | --- | --- | --- | --- |
| 295.1139 | 296.1212 | 7.19 | [M+H]^+^ | 0.806 | C_17_H_17_N_3_S | Quetiapine - C_4_H_8_O_2_ A | 210.0374 | 253.0790 | 221.10659 | 227.0733 | 279.0946 |
| 295.1136 | 296.1209 | 7.61 | [M+H]^+^ | 0.806 | C_17_H_17_N_3_S | Quetiapine - C_4_H_8_O_2_ B | 210.0402 | 253.0789 | 183.02591 | 221.1064 | 227.0649 |
| 311.1090 | 312.1163 | 4.87 | [M+H]^+^ | 0.833 | C_17_H_17_N_3_OS | Quetiapine - C_4_H_8_O A | 226.0319 | 269.0737 | 243.05833 | 295.0891 | 208.0218 |
| 399.1617 | 400.1689 | 5.06 | [M+H]^+^, [M+Na]^+^ | 0.953 | C_21_H_25_N_3_O_3_S | Quetiapine + O A | 269.0745 | 295.0900 | 158.11781 | 226.0381 | 237.1021 |
| 311.1092 | 312.1165 | 5.24 | [M+H]^+^ | 0.824 | C_17_H_17_N_3_OS | Quetiapine - C_4_H_8_O B | 264.1491 | 221.1071 | 195.09177 | 198.0454 | 247.1223 |
| 355.1356 | 356.1427 | 5.37 | [M+H]^+^, [M+Na]^+^ | 0.865 | C_19_H_21_N_3_O_2_S | Quetiapine - C_2_H_4_ A | 221.1069 | 308.1753 | 269.0739 | 247.1227 | 114.0915 |
| 355.1356 | 356.1430 | 5.4 | [M+H]^+^, [M+Na]^+^ | 0.898 | C_19_H_21_N_3_O_2_S | Quetiapine - C_2_H_4_ B | 221.1071 | 308.1750 | 269.08356 | 247.1227 | 114.0915 |
| 399.1616 | 400.1690 | 5.44 | [M+H]^+^, [M+Na]^+^ | 0.919 | C_21_H_25_N_3_O_3_S | Quetiapine + O B | 221.1072 | 352.2015 | 269.07403 | 247.1230 | 158.1255 |
| 415.1568 | 416.1643 | 5.96 | [M+H]^+^, [M+Na]^+^, [M+H]^+^ | 0.977 | C_21_H_25_N_3_O_4_S | Quetiapine + O_2_ A | 207.1020 | 243.0580 | 219.09103 | 285.0687 | 368.1969 |
| 415.1564 | 416.1637 | 6.13 | [M+H]^+^ | 0.949 | C_21_H_25_N_3_O_4_S | Quetiapine + O_2_ B | 207.0921 | 243.0582 | 219.09035 | 285.0702 | 350.1848 |
| 353.1197 | 354.1269 | 7.94 | [M+H]^+^ | 0.888 | C_19_H_19_N_3_O_2_S | Quetiapine - C2H6 A | 253.0790 | 210.0382 | 221.11325 | 279.0944 | 183.0256 |
| 399.1612 | 400.1687 | 5.56 | [M+H]^+^, [M+Na]^+^, [M+Na]^+^, [M+H]^+^, [M+K]^+^ | 0.958 | C_21_H_25_N_3_O_3_S | Quetiapine + O C | 221.1081 | 352.2026 | 269.0742 | 247.1227 | 295.0899 |
| 399.1615 | 400.1688 | 6.69 | [M+H]^+^ | 0.957 | C_21_H_25_N_3_O_3_S | Quetiapine + O D | 221.1067 | 352.2016 | 269.0738 | 247.1238 | 295.0889 |
| 355.1353 | 356.1426 | 8.07 | [M+H]^+^ | 0.902 | C_19_H_21_N_3_O_2_S | Quetiapine - C2H4 C | 210.037 | 221.1067 | 279.0945 | 227.0634 | 239.0630 |
| 311.1087 | 312.1159 | 9.73 | [M+H]^+^ | 0.825 | C_17_H_17_N_3_OS | Quetiapine - C4H8O C | 210.0369 | 227.0633 | 253.0786 | 221.1066 | 294.1055 |
| 337.1246 | 338.1318 | 9.96 | [M+H]^+^ | 0.871 | C_19_H_19_N_3_OS | Quetiapine - C_2_H_6_O | 210.0365 | 296.1200 | 253.0784 | 305.2116 | 320.1220 |
| 415.1565 | 416.1638 | 4.49 | [M+H]^+^ | 0.977 | C_21_H_25_N_3_O_4_S | Quetiapine + O_2_ C | 237.1020 | 368.1966 | 285.0686 | 263.1177 | 158.1178 |
| 415.1568 | 416.1641 | 4.56 | [M+H]^+^ | 0.944 | C_21_H_25_N_3_O_4_S | Quetiapine + O_2_ D | 237.1020 | 368.1966 | 285.0686 | 263.1177 | 158.1178 |
| 355.1353 | 356.1425 | 4.92 | [M+H]^+^ | 0.905 | C_19_H_21_N_3_O_2_S | Quetiapine - C_2_H_4_ D | 269.0745 | 226.0365 | 114.0938 | 237.1055 | 295.0935 |
| 397.1457 | 398.1530 | 5.04 | [M+H]^+^ | 0.94 | C_21_H_23_N_3_O_3_S | Quetiapine + O - H_2_ A | 398.1530 |  |  |  |  |
| 415.1565 | 416.1638 | 5.39 | [M+H]^+^ | 0.981 | C_21_H_25_N_3_O_4_S | Quetiapine + O_2_ E | 226.0365 | 237.1020 | 295.0915 | 368.1965 | 255.0605 |
| 311.1086 | 312.1159 | 5.52 | [M+H]^+^ | 0.835 | C_17_H_17_N_3_OS | Quetiapine - C_4_H_8_O D | 198.0361 | 295.0954 |  |  |  |
| 415.1563 | 416.1636 | 6.07 | [M+H]^+^ | 0.949 | C_21_H_25_N_3_O_4_S | Quetiapine + O_2_ F | 285.0685 | 311.0842 | 354.1276 | 207.0917 | 158.1190 |
| 381.1508 | 382.1581 | 6.76 | [M+H]^+^ | 0.922 | C_21_H_23_N_3_O_2_S | Quetiapine - H_2_ | 279.0945 | 253.079 | 314.1309 | 221.1063 | 130.0856 |
| 339.1406 | 340.1479 | 7.35 | [M+H]^+^ | 0.879 | C_19_H_21_N_3_OS | Quetiapine - C_2_H_4_O A | 253.0792 | 221.1161 | 279.0946 | 210.0369 | 114.0916 |
| 339.1394 | 340.1467 | 7.6 | [M+H]^+^ | 0.881 | C_19_H_21_N_3_OS | Quetiapine - C_2_H_4_O B | 325.0783 | 279.0936 | 114.0908 | 210.0373 | 221.1065 |
| 397.1457 | 398.1533 | 7.82 | [M+H]^+^, [M+Na]^+^ | 0.944 | C_21_H_23_N_3_O_3_S | Quetiapine + O - H_2_ B | 253.0788 | 279.0946 | 221.1068 | 172.0967 | 210.0367 |
| 369.1144 | 370.1217 | 8.61 | [M+H]^+^ | 0.908 | C_19_H_19_N_3_O_3_S | Quetiapine-M (N-CH_2_-COOH-HO-piperazine) | 210.0377 | 221.1064 | 227.0646 | 322.1554 | 253.0805 |
| 369.1146 | 370.1218 | 5.7 | [M+H]^+^ | 0.906 | C_19_H_19_N_3_O_3_S | Quetiapine-M (N-CH_2_-COOH-sulfoxide) | 221.1066 | 322.1541 | 247.1202 | 269.0726 | 195.0908 |
